# The amorphization of crystalline silicon by ball milling

**DOI:** 10.1016/j.heliyon.2024.e34881

**Published:** 2024-07-19

**Authors:** Roby Gauthier, B. Scott, J. Craig Bennett, Mina Salehabadi, Jun Wang, Tariq Sainuddin, M.N. Obrovac

**Affiliations:** a Department of Chemistry, Dalhousie University, Halifax, N.S, B3H 4R2, Canada; b Department of Physics, Acadia University, Wolfville, N.S, B4P 2R6, Canada

## Abstract

The transformation of crystalline silicon to amorphous silicon during ball milling was quantitatively measured by x-ray diffraction and electrochemical methods. Amorphous silicon was found to form rapidly from the very initial stages of ball milling. Simultaneously, the grain size of the crystalline silicon phase decreased. Under extended milling times it was found that a maximum of 86 % of the silicon became amorphous. Similarly, the grain size of the crystalline silicon phase could not be reduced below 6 nm. This transformation followed an Avrami kinetic model, which is consistent with a system which reaches a steady state. These observations suggest a mechanism in which ball milling generates defects, resulting in silicon amorphization and grain size reduction, where the degree of amorphization is limited in extent because there exists a limiting silicon grain size below which defects are no longer formed.

## 1 Introduction

Due to silicon's natural abundance and large volumetric capacity for lithium, the use of silicon-based anode materials for Li-ion batteries has garnered great interest [1,2]. For this application, nanocrystalline or amorphous silicon in the form of Si nanoparticles or nanograins of Si in SiO or Si-alloys, is preferred over crystalline silicon due to their improved cycle life [[3], [4], [5], [6], [7], [8], [9], [10]]. For instance, greatly improved cycling performance has been observed when using nanocrystalline Si made by ball milling compared to crystalline Si [11,12]. This can be explained by the more homogeneous swelling and reduced fracture during cycling of nanocrystalline silicon particles versus crystalline silicon particles [11]. However, in commercial Li-ion cells, amorphous or nanocrystalline Si is not typically used in its pure form, but is usually further processed, such as in Si–C composite materials [[2], [3], [4]].

As an industrially scalable process, ball milling is an important method of making nanocrystalline and amorphous silicon. At the lab-scale ball milling is typically conducted in so-called high energy ball mills (HEBM) e.g. a SPEX mill or in planetary mills [[11], [12], [13], [14]]. Here we define HEBM as those milling conditions with impact energies >20 J. For instance, SPEX milling is typically conducted with 0.25" - 0.5 " (6.35–12.7 mm) steel balls, with balls impacting together each with speeds of about 4 m/s [15], corresponding to impact energies of ∼30–270 J. Low energy ball milling processes (LEBM), such as horizontal ball milling on laboratory-scale have impact energies that are <10 mJ (e.g. ∼ 2 mJ for a 6" (15 cm) diameter mill with 6.35 mm steel balls [15,16]). Such low impact energies can also be achieved in a SPEX mill if the ball size is reduced (e.g. ∼4 mJ for 3 mm steel balls) [17]. Although amorphization during ball milling, including the amorphization of Si, is associated with higher impact energy [18], we have found that the opposite is the case [17]. Indeed, the highest degree of amorphization can be achieved by LEBM, for instance in low energy roller mills or in a SPEX mill utilizing small (<3 mm) ball diameters [17]. Due to the large variation in milling conditions in different studies, the ball milled Si products have also varied [11,12,[17], [18], [19]]. Nevertheless, most studies observe a reduction in Si grain size and Si amorphization during the ball milling process. Notably, Yu et al. measured the amorphous fraction of ball milled Si at different milling times by deconvolution of x-ray diffraction patterns, but no attempt was made to identify or model trends in structural evolution [12]. Shen et al. used HEBM (SPEX mill with 6–8 mm diameter steel balls) and observed the evolution of Si during ball milling. Under these conditions only 57 % of the Si could be converted to the amorphous phase after 20 h of milling, with the remaining c-Si being in the form of small (3–20 nm) crystallites [19].

Amorphization of silicon during ball milling has been explained to occur via multiple mechanisms. Shen et al. suggested that Si amorphization might proceed due to the high local pressures imparted during HEBM (3.30–6.18 GPa) followed by rapid decompression [20]. S. Zhao et al. used laser pulse experiments to induce Si amorphization by shock compression. Si amorphization was found to occur above a shock pressure threshold of 11.2 GPa, with amorphous Si (a-Si) nucleating at crystal defects near the point of applied shock compression at the surface and propagating towards the bulk of the sample [21]. However, this model does not account for the observation that Si amorphization occurs more readily by LEBM [17]. Y.H. Zhao et al. showed that Se amorphized during ball milling via a destabilization model, where grains spontaneously amorphize once they are reduced to a critical size [22]. Similarly, Shen et al. [20] and Gaffet et al. [23] suggested that as the Si grain size is reduced during ball milling, a critical size is reached below which the diamond lattice becomes unstable. Huang et al., proposed that silicon amorphization by ball milling was caused by the creation of crystalline defects that result in an elevation of energy, ultimately leading to the transition from the crystalline Si (c-Si) to the a-Si phase [24]. Concurrently, the creation of defects causes the c-Si to reduce in size during ball milling until strain is relieved in grain boundaries, instead of by the creation of new defects. This results in the formation of a nano-crystalline/amorphous Si composite. Similarly, Mirabootalebi proposed that Si amorphization proceeds from crystallite surfaces [14].

Numerous models have been used to study the kinetics of the ball milling process [[25], [26], [27]]. As one example, the Avrami equation has been successfully used to model the kinetics of material evolution during ball milling. This includes modelling aspects of the Si ball milling processes, including how the specific surface area of Si evolves [26] and how Si reacts with gasses during ball milling [27]. In both these cases, the rates of these processes were found to be proportional to the fraction of “destructible” Si particles in the sample, while the "indestructible" Si particles were those that were found to be too small to fracture (∼10 nm). This behavior is consistent with the model proposed by Huang et al. mentioned above.

Despite the many previous studies, we are not aware of any that propose a kinetic model to describe the transformation of c-Si to a-Si during ball milling. This would be highly useful in the understanding of the process and its optimization. In addition, most studies use ball milling conditions that are far from practical (e.g. amorphization achieved only after tens of hours of milling). Therefore, their applicability to practical Si amorphization is questionable. In this study, the transformation of c-Si to a-Si is quantitatively studied using practical milling conditions under which Si is rapidly and efficiently amorphized (within minutes) [17]. For the first time we also present a quantitative measure of this transformation in terms of c-Si crystallite size, and the amount of a-Si formed during milling as measured by XRD and by electrochemical means. Using these observations, a kinetic model is developed to describe the transformation of c-Si to a-Si. This study enables a clearer understanding of the formation mechanism of a-Si from c-Si during practical ball milling conditions. The kinetic model developed for this transformation could further help to optimize the amorphization process.

## 2 Experimental

Ball milled silicon was prepared by adding 1.165 g of crystalline silicon powder (Skyspring Nanomaterial, 99.999 % metal basis, ∼325 mesh) to ball milling containers (SPEX CertiPrep, 65 ml hardened steel) with 180 g of stainless-steel balls (d = 1/16 inch), corresponding to a 155:1 ball to powder ratio by mass and a ∼48:1 ball to powder ratio by volume. These conditions were optimized in a previous study to provide fast amorphization while minimizing iron contamination [17]. The containers were sealed in an argon atmosphere and then placed in a high energy ball mill (SPEX CertiPrep 8000) for specific amounts of time (0.05–32 h). To collect the samples, approximately 20 mL of ethanol was added to the milling vials and the contents were then ball milled for an additional 10 min in air. The resulting silicon/ethanol slurry was then separated from the milling balls using a sieve. The recovered slurry was then dried in a solvent oven at 120 °C in air for ∼2 h and then hand ground using a mortar and pestle.

Oxygen contents were determined by the LECO method (NSL Analytical Services, Inc., Cleveland OH) to an error within 5 % of its reported value. X-ray diffraction (XRD) patterns were collected using a Rigaku Ultima IV diffractometer equipped with a Cu Kα X-ray source (λ = 1.5406 Å), a diffracted beam monochromator, and a scintillation counter detector. Data was collected in the 2*θ* = 10–80° range in 0.05° increments with 8 s per step. Scanning electron microscopy (SEM) images were obtained with a JEOL JSM-IT200 microscope using a 5 kV or 10 kV accelerating voltage. Transmission electron microscopy (TEM) images were obtained using a Philips CM30 TEM. TEM specimens were made by suspending sample powders in methanol, sonicating for 5 min, and placing a drop onto a lacey carbon-coated TEM grid.

Powder patterns of small Si clusters were modeled using the Debye scattering equation:

(1) $$I=\sum_{m}\sum_{n}f_{m}f_{n}\frac{\sin\left(k\cdot r_{\text{mn}}\right)}{k\cdot r_{\text{mn}}}$$

where I is the x-ray scattering intensity, f is the atomic scattering factor, k = (4πsin*θ*)/λ and r_mn_ is the distance between atoms m and n. Two types of Si clusters were considered for this model: consisting either of a 1 × 1 × 1 or a 2 × 2 × 2 array of Si unit cells having the diamond lattice structure.

Silicon anode coatings were made from slurries having a 0.76:0.05:0.18 ratio by mass of silicon:carbon black (Super C65, Imerys Graphite and Carbon):lithium polyacrylate (from a polyacrylic acid solution (10 wt% in H_2_O, average molecular weight 250,000 g/mol, Sigma-Aldrich) neutralized with LiOH·H_2_O (98 %, Sigma-Aldrich) in distilled water). A few drops of isopropanol was added to each slurry to decrease the surface tension of water. The slurry was then mixed with a high shear mixer with a Cowles blade for 10 min before coating onto copper foil using a 4/1000-inch (∼100 μm) stainless steel coating bar. The active loading of the resulting electrodes was ∼0.8 mg/cm^2^. The coatings were then dried at 120 °C for 1 h, punched into 13 mm disk electrodes and vacuum dried at 120 °C overnight.

Electrolyte solutions were made by dissolving 1 M LiPF_6_ in a 3:6:1 ratio by mass of ethylene carbonate, diethyl carbonate and fluoroethylene carbonate (all battery-grade from BASF). Half-cells were built using 2325-type coin cell hardware in an argon filled glovebox. The cell stack consisted of: the working electrode, a blown microfiber (BMF, 3 M) separator, a Celgard-2300 separator, a lithium metal foil (99.9 %, Sigma Aldrich) counter/reference electrode, and a Cu-spacer. Electrochemical testing of silicon half-cells was performed at 30 °C using a Maccor Series 4000 Automated Test System (Maccor Inc., Tulsa OK). For these tests, cells were discharged (lithiation) and charged (delithiation) between 5 mV and 0.9 V at a constant current of C/20 to 50 mV. At the end of every discharge, cells were held at 5 mV until the current decreased to C/40. a-Si cells were cycled at a current of C/40 rate. Here C-rate is defined as the current required to fully lithiate Si in 1 h, based on its theoretical capacity of 3578 mAh/g [28].

## 3 Results and discussion

As received, the crystalline Si had a measured oxygen content of 8.1 ± 0.4 atomic %. After ball milling the crystalline Si for 20 h in argon, collecting the ball milled Si by further milling in ethanol, and then heating the collected Si in air at 120 °C, the oxygen content was measured to be 7.9 ± 0.4 atomic % (within error of the unmilled sample). Therefore, within measurement error, no oxygen absorption could be detected as a result of the ball milling process or subsequent air exposure. All samples therefore have an oxygen impurity of about 8 atomic %. This low oxygen level was also confirmed electrochemically, as discussed later in this article.

**Fig. 1:**
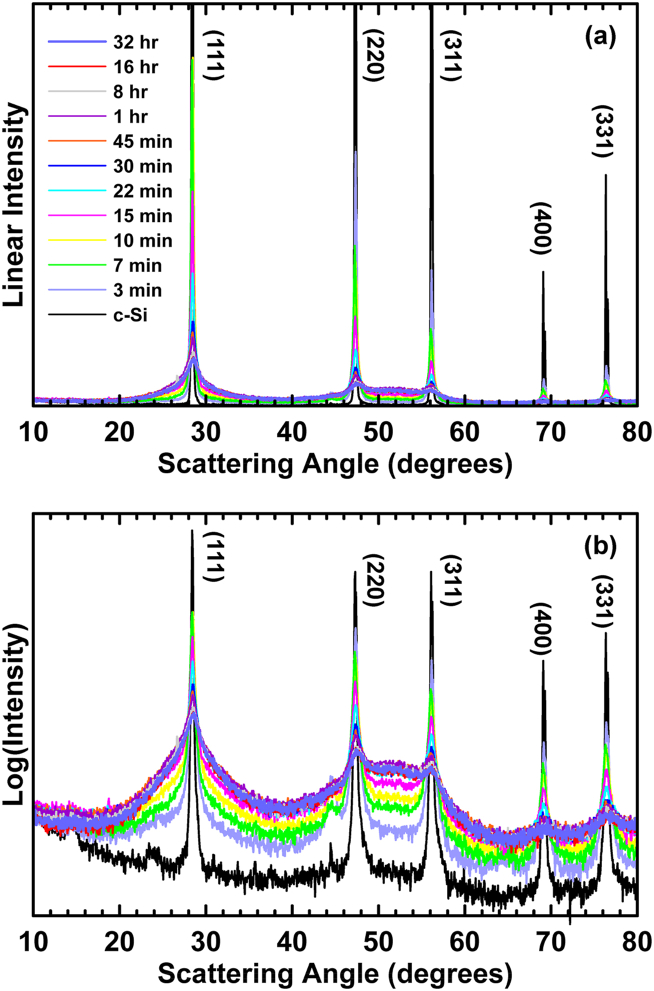
XRD patterns of c-Si ball milled for different amount of time from 0 min to 32 h plotted with a (a) linear and (b) logarithmic intensity scale.

Fig. 1(a) shows XRD patterns of crystalline Si ball milled for different times. To better visualize changes during the ball milling process, Fig. 1(b) shows the same XRD patterns plotted on a logarithmic intensity scale. On this log scale small features are visible in the XRD pattern of unmilled Si. Peaks near 13° and 24° in this pattern are thought to be due to silicon hydroxides and oxides, respectively. These compounds are known to be the surface species on silicon and many phases of these compounds exist with peaks in this region. Small peaks near 44° are due to Fe contamination from the milling vial and media. We have determined from an earlier study that the amount of iron contamination from ball milling Si under identical conditions for up to 16 h is 1.12 ± 0.04 at.% [29]. A very broad peak is apparent in the unmilled Si XRD pattern at about 50°. Such broad peaks are typical of amorphous phases [30]. The appearance of broad peaks in the unmilled Si XRD pattern may be due to a small amount of amorphous Si already being present in this sample. Within the first 3 min of ball milling broad amorphous-like peaks are readily apparent in the XRD pattern. As ball milling is continued the broad amorphous Si peaks increase in intensity at the expense of the intensity of the sharper crystalline peaks. This transformation is rapid, with drastic changes in the Si XRD patterns already occurring within 15 min of ball milling. After about 1 h of ball milling the XRD patterns start to converge.

**Fig. 2:**
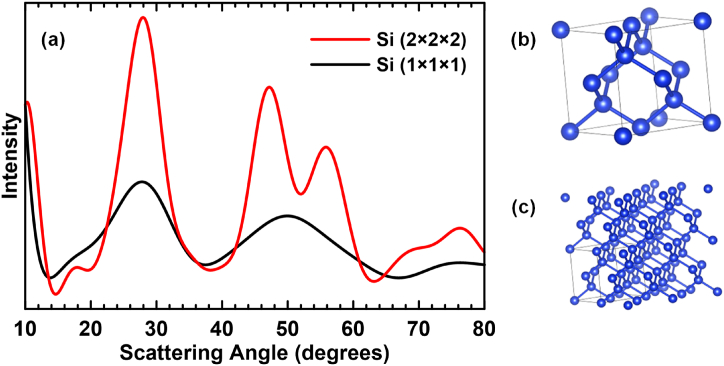
(a) Calculated XRD patterns using the Debye scattering equation of a single Si unit cell (as shown in (b)), and a 2 × 2 × 2 cluster of Si unit cells (as shown in (c)).

To quantitatively determine the amount of amorphous Si phase that forms over time during ball milling, the XRD patterns in Fig. 1 were fit numerically. As an initial attempt, the XRD patterns were fit using pseudo-Voigt peak functions for the c-Si phase and for the a-Si phase according to the known peak positions for c-Si. While good fits could be obtained for the c-Si phase, this method did not fit the a-Si phase well at angles between 40° and 65°. In this region, the a-Si phase XRD pattern appears to be composed of a single peak centered at about 52°. If pseudo-Voigt peaks representing the Si (220) peak at 47° and the Si (311) peak at 56° are simply broadened according to the Scherrer formalism, they do not form this observed peak shape. The reason for this has been demonstrated previously by considering diffraction from very small (few unit cells) clusters of Si atoms [31,32]. In such cases the Si (220) and (311) peaks merge into a single peak. This is illustrated in Fig. 2, which shows Debye calculations for a 2 × 2 × 2 and a 1 × 1 × 1 cluster of Si unit cells. The Si (220) and (311) peaks are still apparent for the 2 × 2 × 2 cluster, but only a single peak centered roughly between the Si (220) and (311) peaks is present for the 1 × 1 × 1 cluster. This shows that ordering in ball milled amorphous Si is over a very short range that is about the size of a Si unit cell (∼5.4 Å).

Replacing the individual Si (220) and (311) peaks with a single pseudo-Voigt peak, according to the Debye model results, yielded excellent fits for the ball milled Si XRD patterns. The series of XRD patterns shown in Fig. 1 was fit as follows. First a background function was established by fitting the unmilled Si pattern with pseudo-Voigt peaks for the crystalline phase only, while the background was modeled as:

(2) $$background=B_{0}+B_{1}\left[\frac{1+\cos^{2}\;2\theta }{\sin\;\theta \;\sin\;2\theta }\right]$$

**Fig. 3:**
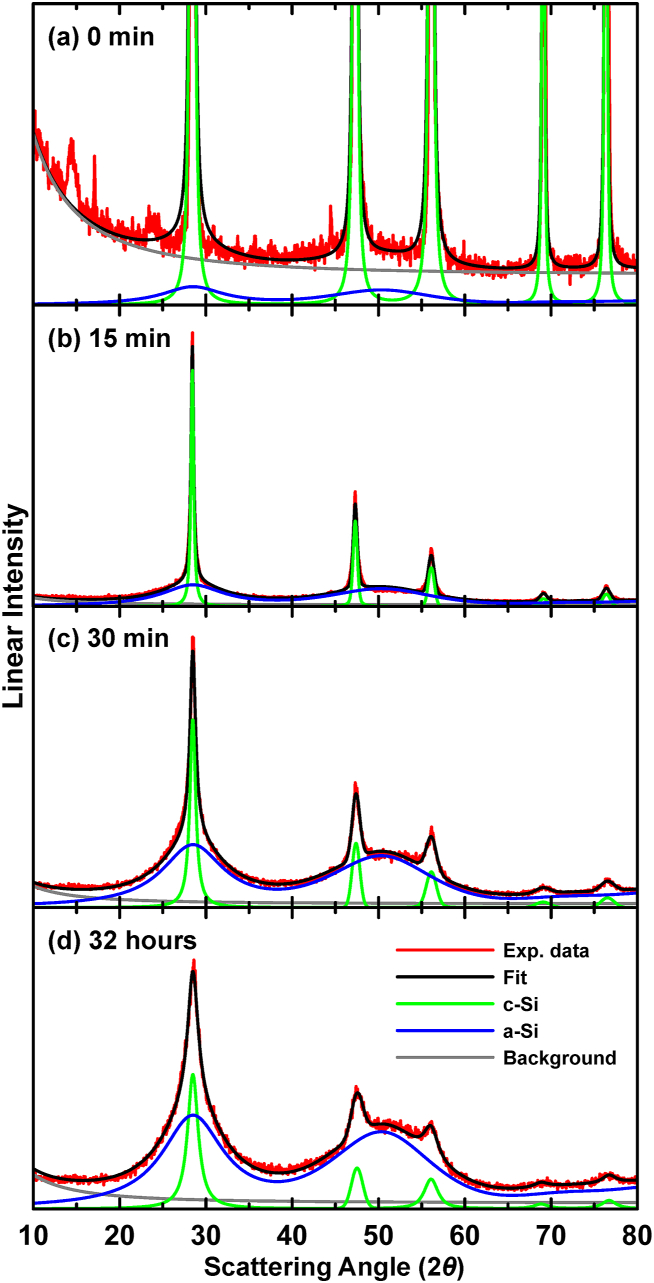
Selected fits of XRD patterns of c-Si samples after ball milling for (a) 0 min, (b) 15 min, (c) 30 min, and (d) 20 min.

Where 2*θ* is the scattering angle, the term in square brackets is the Lorentz-polarization factor for powder patterns [33], and *B*_*0*_ and *B*_*1*_ are fitting parameters representing constant background radiation and the unmodified scattered intensity, respectively. This function fits the background of the unmilled Si sample well, as shown in Fig. 3(a). With the background function established, the same values of *B*_*0*_ and *B*_*1*_ were then used to fit all of the other XRD patterns. The sample that was ball milled for the longest time (32 h) was then used to establish the fitting function for the a-Si phase. For this phase, pseudo-Voigt peaks were used to fit all the Si XRD peaks, excepting that only one peak was used to fit the Si (220) and (311) peaks, according to the Debye model results. The c-Si phase was fit with pseudo-Voigt peaks for all the Si peaks. Fig. 3(d) shows this fit.

To fit all of the XRD patterns, the established background function was used and to this the function of the a-Si phase was added, with only the amplitude of this function as an adjustable parameter. The c-Si phase was modeled with pseudo-Voigt peaks, with the relative amplitude and breadth of each peak allowed to vary. This resulted in excellent fits for all the XRD patterns. Selected fits are shown in Fig. 3(a–d) for those samples ball milled for 0 min, 15 min, 30 min, and 32 h, respectively. Fig. 3(a) shows that even the unmilled Si sample had some a-Si component. As the milling time is increased, the a-Si phase grows at the expense of the c-Si phase. The c-Si phase XRD peak widths also broaden with milling time. These results show that the a-Si phase forms quickly and is unchanged during the milling process, while the c-Si phase grain size becomes smaller.

**Fig. 4:**
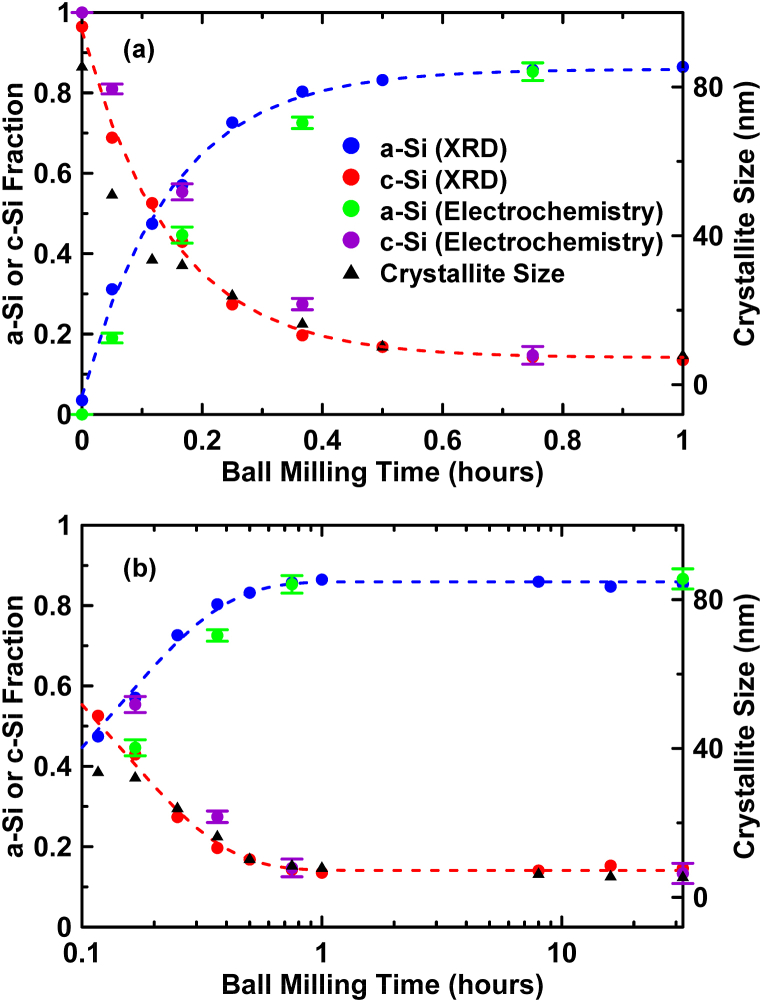
The relative amount of a-Si and c-Si phases in ball milled Si samples vs. the ball milling time as calculated from XRD patterns. Fits to the a-Si and c-Si phase fractions according to the Avrami kinetic equation are shown as dashed lines. Additionally shown are a-Si and c-Si phase fractions as determined by electrochemistry and the c-Si grain size as a function of milling time. These data are plotted vs. a (a) linear and (b) logarithmic time scale.

Fig. 4(a) and (b) show plots on a linear and logarithmic time scale, respectively, of the amounts of c-Si and a-Si phases present as a function of milling time, based on the relative total XRD peak areas for each phase. The fraction of a-Si phase present as a function of milling time was fit by least squares fitting using the Avrami equation with an Avrami exponent equal to one:

(3) $$A\left(t\right)=A_{\lim}-\left(A_{\lim}-A_{0}\right)e^{-Kt}$$

where *t* is the milling time, *A*(*t*) corresponds to the fraction of a-Si present at time *t*, *A*_0_ is the initial amount of a-Si at time *t* = 0, *K* is the rate at which a-Si is being created, and *A*_*lim*_ is the amount of amorphous phase when *t* tends toward infinity. A good fit was obtained using this model with A_lim_ = 0.86, A_0_ = 0.05 and K = 6.79 h^−1^. The amount of c-Si phase can be deduced using 1 - *A*(*t*). Also shown in Fig. 4 is an estimate of the c-Si grain size as a function of milling time, based on applying the Scherrer equation to the c-Si (111) peak. The grain size also roughly follows the Avrami model, with a limiting grain size of about 9 nm.

A model equivalent to the Avrami model has been used to describe the reaction of Fe and Cu during ball milling [25]. The Avrami model has also been used to describe the change in surface area of powders during ball milling [26]. We have also previously used this model to successfully describe the reaction between Si and CO_2_ during ball milling [27]. In the latter study, it was found that the Avrami model described the formation of fresh Si surfaces during ball milling, which then immediately reacted with CO_2_. The reaction could not proceed further when the size of the Si could not be reduced further, so that no new fresh surfaces were created. The size of these "indestructible particles" was found to be 10 nm, which is a grain size where most materials encounter Hall-Petch breakdown. That is, below this grain size stress results in deformations along grain boundaries rather than the creation of new defects/fractures. In the present study, the observed Avrami kinetic behavior in the grain size also verifies that the formation of a-Si stops when a similar limiting Si grain size is reached.

These results are consistent with the mechanism proposed by Huang et al., in which a-Si forms at dislocations formed within c-Si grains during ball milling [24]. According to this mechanism, a-Si is formed at defects during ball milling, resulting in c-Si grains becoming progressively smaller. This continues until a limiting grain size is reached corresponding to the Hall-Petch breakdown size. The speed of this reaction in Si is remarkable. In our previous study of the reactivity of Si with CO_2_ during ball milling, the rate constant *K* for this reaction was found to be 1.65 h^−1^ [27]. This is much smaller than the rate constant of 6.79 h^−1^ found here for the amorphization of c-Si. This result is understandable, since for reactive gas milling, the rate refers to the exposure of fresh surfaces of Si to the reactive gas. Here, the rate is related to the creation of dislocation sites throughout the entire material, which will be much more prevalent than the creation of new exposed surfaces.

**Fig. 5:**
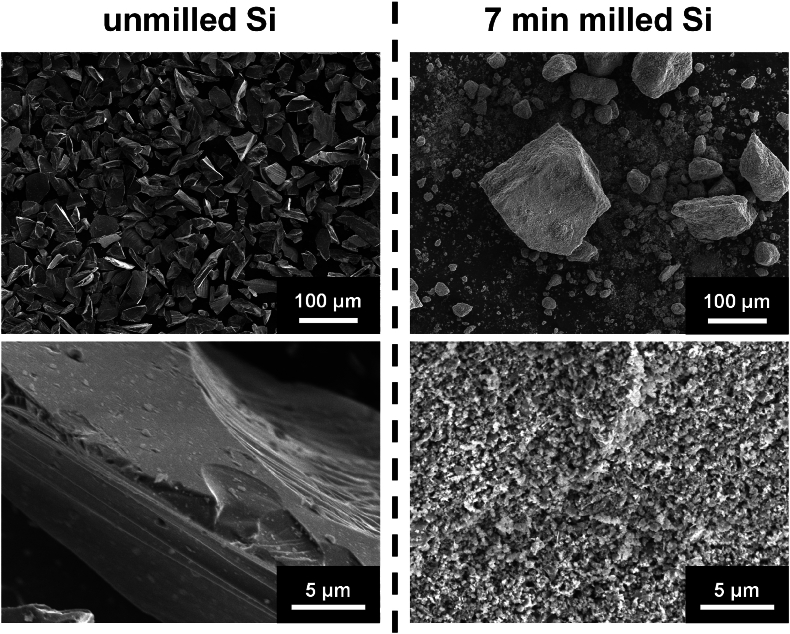
SEM images of pristine c-Si and c-Si after ball milling for 7 min at two different magnifications.

To gain more insight about the ball milling process, the ball milled powders were studied by electron microscopy. Fig. 5 shows SEM images of the unmilled Si powder and of Si powder ball milled for 7 min. The unmilled Si powder consists of ∼50 μm irregularly shaped particles with smooth surfaces (∼1 m^2^/g surface area). After 7 min of ball milling the Si has been transformed into ∼5–100 μm secondary particles (∼30 m^2^/g surface area). The morphology and surface area of samples milled for longer times were similar. These secondary particles are loose agglomerates of primary particles that are less than 0.5 μm in size. This agglomeration is an artifact of the method that was used to recover the ball milled Si powder. That the Si powder has been completely transformed into <0.5 μm particles after only 7 min is another indication of the rapid transformations that occur during the ball milling process. No differences could be seen by SEM between the 7 min milled sample and samples that had been milled longer.

**Fig. 6:**
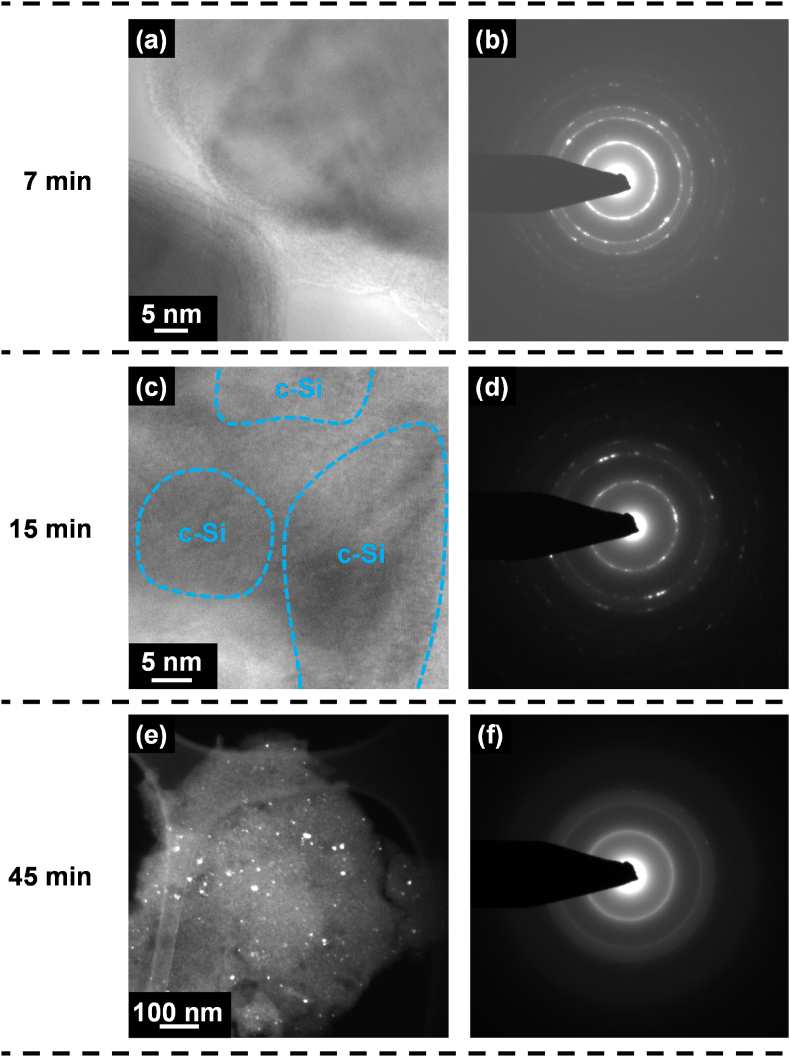
TEM images and a SAEDPs of c-Si after ball milling for (a,b) 7 min, (c,d) 15 min, and (e,f) 45 min. (a) and (c) are BF images. (e) is a DF image centered on a segment of the Si(111) reflection ring. In panel (c), dashed lines indicate regions containing parallel lattice fringes corresponding to the d-spacing of the (111) Si lattice planes (0.313 nm).

Further insight in the evolution of Si during ball milling was obtained from TEM studies. These studies showed that most of the Si sample ball milled for 7 min consisted of primarily c-Si particles surrounded with a-Si at their edges. This can be clearly seen in Fig. 6(a), which shows a TEM image of two particles. Moire patterns and lattice fringes can be seen in the bulk of the particles, but the edges of these particles are primarily composed of a-Si. Because of the large particle size reduction that occurred after 7 min of milling, these amorphous particle edges may be associated with fracture sites. This supports the idea that a-Si is formed at defect sites. Selected area electron diffraction patterns obtained for the Si sample ball milled for 7 min showed that the degree of crystallinity of the particles was generally substantially impacted. Fig. 6(b) shows the SAEDP observed for many of the fragments. The ring pattern was indexed to Si and is consistent with many small crystallites (ring pattern) mixed with fewer larger crystallites (spots).

Fig. 6(c) shows a TEM image of the Si sample ball milled for 15 min. The sample is now mostly amorphous with small c-Si regions, which is consistent with XRD results. Highlighted in the image are c-Si regions as indicated by areas of consistent lattice fringes. In this particle, the c-Si regions are ∼15–35 nm in extent, also consistent with the 25 nm average grain size measured by XRD. The SAEDP of this sample, shown in Fig. 6(d) has diffuse rings superposed with the rings of c-Si, indicating that much of the sample is amorphous. From TEM studies, the 45 min milled sample consisted of small (5–10 nm) c-Si particles embedded in an amorphous Si matrix. This is most easily visualized in the dark-field image shown in Fig. 6(e), which was formed with the objective aperture centered on a segment of the (111) Si ring. In this image the small c-Si particles diffracting strongly to this location appear bright. Fig. 6(f) shows an SAEDP from this sample. In this pattern diffuse rings, indicative of a-Si, are observed superposed with broadened rings from c-Si indicating the presence of Si nanocrystals. Overall, TEM observations are consistent with XRD results, while showing the formation of a-Si at fracture zones and that the structure of the samples is one of c-Si nanocrystals dispersed in an a-Si matrix.

**Fig. 7:**
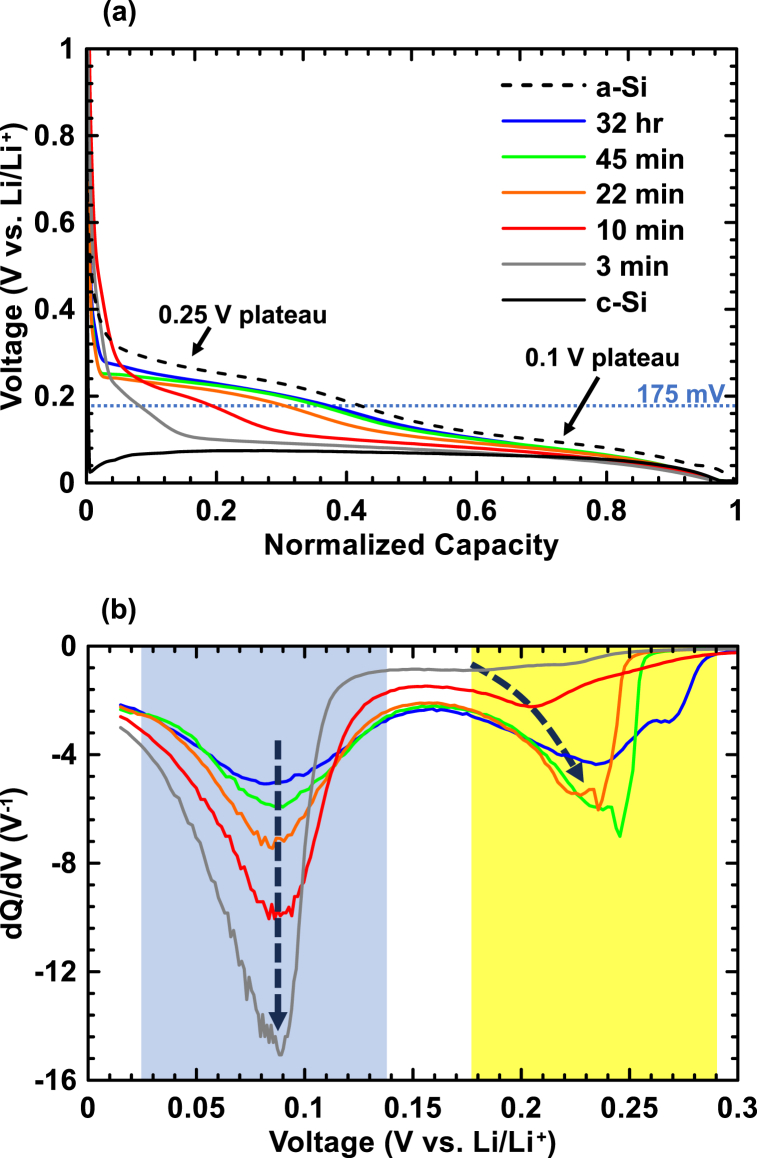
(a) Voltage versus normalized capacity and (b) differential capacity versus voltage curves of silicon half-cells. Dashed arrows in (b) show trends in the differential capacity peak potentials as the ball milling time is increased. The peaks near 0.1 V are highlighted in blue and the peaks near 0.25 V are highlighted in yellow.

Further verification of the c-Si to a-Si transition during ball milling was obtained from the electrochemical lithiation characteristics of the ball milled samples as measured in lithium half cells. All cells reached a full lithiation capacity close to the theoretical value for the formation of Li_15_Si_4_ (3578 mAh/g) [28], within a standard deviation of 8 %, due to weighing error and capacity from SEI formation. To eliminate this random error, the voltage curves were normalized with respect to their capacity at full lithiation. Fig. 7(a) shows a plot of the voltage versus normalized capacity of the first lithiation of ball milled Si samples. Also shown are the voltage curves for the lithiation of c-Si and a-Si. Corresponding differential capacity curves are shown in Fig. 7(b). The voltage of the c-Si cell initially drops to 5 mV due to slow kinetics from the initial nucleation and growth of lithiation sites. After this, c-Si lithiates in a 2-phase region, here at a constant voltage of about 80 mV. Careful studies at slower rates have shown that c-Si lithiates at 170 mV [34]. This potential is indicated by a dotted line in Fig. 7(a). The voltage curve of a-Si was obtained by fully lithiating and then delithiating c-Si which is known to result in the full amorphization of Si [28,34]. The a-Si lithiation voltage curve shown is from the third lithiation half-cycle. A small initial plateau at about 0.4 V is due to oxygen content in ball milled Si, as previously reported in Ref. [35]. This plateau is small, in agreement with the small amount of oxygen in these samples. The size of this plateau compared to the total discharge capacity can be correlated to the total oxygen content in ball milled Si [35]. For these samples, the 0.4 V plateau corresponds to less than 4 % of the total discharge capacity, which corresponds to an oxygen content of about 7 atomic %. This electrochemical result is close to the direct oxygen content measurement of about 8 atomic % for these samples. Subsequent to the small high voltage oxygen plateau, a-Si lithiates. The a-Si lithiation voltage curve is very different than that of c-Si [28,34,36]. Lithiation of a-Si begins at a much higher voltage, with the voltage curve consisting of two sloping plateaus. These are manifest as two peaks in the differential capacity curve at about 0.1 V and 0.25 V, which are highlighted in blue and yellow, respectively in Fig. 7(b).

As shown in Fig. 7(a)–as the ball milling time is increased, the 0.25 V a-Si plateau correspondingly increases, commensurate with increased formation of a-Si. This effect has been previously observed for ball milled Si [12]. The increasing amount of a-Si can also be seen in the differential capacity curves in Fig. 7(b) as an increase in the 0.25 V peak area with milling time. The 0.1 V a-Si plateau is coincident with the lithiation plateau of c-Si and therefore is not easy to detect in the voltage or differential capacity curves. To quantitatively determine how much a-Si is in each of these samples, it was first determined that the portion of the pure a-Si voltage curve that appears above 173 mV (i.e. the a-Si upper voltage plateau, at potentials above the c-Si lithiation potential) accounts for 44 % of the total a-Si capacity. From this value, the total capacity contribution from the a-Si phase could be calculated from each sample's capacity above 173 mV.

The results of these calculations are shown in Fig. 4. There is good agreement with XRD results for samples milled 45 min or more. For those samples milled with shorter times, the amount of a-Si is underestimated. The reason for this can be understood by considering, as an example, the voltage curve shown in Fig. 7(a) of the cell with the 10 min milled sample. According to XRD, this sample contains 57 % a-Si and 43 % c-Si. The cell shown was discharged at a C/20 rate. As this cell is discharged only the a-Si phase initially begins to lithiate, while the c-Si phase initially remains inactive. Since the a-Si phase accounts for 57 % of the capacity, this means that the a-Si phase is being discharged at a C/11 rate on its high voltage plateau. The higher discharge rate being applied to this phase will have the effect of depressing the a-Si high voltage plateau to lower voltages. When the lithiation continues and the potential becomes lower than 170 mV, both the a-Si and c-Si phases lithiate simultaneously and the effective discharge rate becomes C/20 for both phases. Therefore, the lower potential plateau is unaffected. This effect can be seen in the differential capacity curves, shown in Fig. 7(b), where the high voltage plateau increases in potential as the milling time is increased, while the low voltage plateau potential remains the same. As a consequence of the depression of the high voltage plateau, the capacity of this plateau is lessened above 173 mV and the calculated amount of a-Si is correspondingly decreased, as is observed in Fig. 4. Taking into account this polarization effect, the electrochemical results further verifies the evolution in Si microstructure during ball milling as observed by XRD and additionally shows that the amount of a-Si can be quantitatively determined electrochemically.

## 4 Conclusions

In this work, the kinetics and mechanism of c-Si amorphization by low energy ball milling were investigated. During ball milling the initially ∼50 μm crystalline Si particles were rapidly reduced in size to ≤1 μm primary particles. a-Si formation started immediately during the milling process. TEM studies showed that the a-Si initially forms on the fractured surfaces of the Si particles. With continued ball milling, the conversion of a-Si continues until a steady-state a-Si concentration of 86 % is reached after 30 min of ball milling. The size of the c-Si also reduces during ball milling and reaches a steady-state of about 6 nm. TEM studies show that after long milling times, ball milled Si consists of 5–10 nm c-Si grains dispersed in an a-Si matrix. The amount of c-Si converted to a-Si during ball milling was quantitatively determined by XRD measurements. It was found that the amount of a-Si in the samples could also be quantitatively determined electrochemically from the high voltage plateau capacity during the lithiation of a-Si. From these quantitative measurements, it was found that the conversion kinetics of c-Si to a-Si during ball milling followed the Avrami equation. These observations are consistent with the formation of a-Si on defect sites created by ball milling, with a concurrent reduction in c-Si grain size. A steady-state is reached during ball milling when the size of the c-Si particles approaches the Hall-Petch breakdown limit, at which point defects no longer occur within the c-Si particles and a-Si formation stops. These results create a deeper understanding of the amorphization of Si during ball milling and a provide quantitative model to predict its behavior. In addition, it was shown that low energy milling conditions lead to rapid amorphization of Si. We believe these results will be highly valuable for the development of production methods for amorphous and nanocrystalline Si, which have become important materials for Li-ion batteries.

## Data availability

The data associated with this study have not been deposited into a publicly available repository. Data will be made available upon request.

## CRediT authorship contribution statement

**Roby Gauthier:** Writing – review & editing, Writing – original draft, Methodology, Investigation, Formal analysis. **B. Scott:** Methodology, Investigation, Formal analysis, Data curation, Conceptualization. **J. Craig Bennett:** Writing – review & editing, Methodology, Investigation, Formal analysis. **Mina Salehabadi:** Investigation, Formal analysis. **Jun Wang:** Investigation. **Tariq Sainuddin:** Investigation. **M.N. Obrovac:** Writing – review & editing, Writing – original draft, Validation, Project administration, Methodology, Investigation, Funding acquisition, Formal analysis, Conceptualization.

## Declaration of competing interest

The authors declare that they have no known competing financial interests or personal relationships that could have appeared to influence the work reported in this paper.
