# Supplementary material for: The amorphization of crystalline silicon by ball milling
Source: Heliyon. 2024 Jul 19;10(15):e34881. doi: 10.1016/j.heliyon.2024.e34881 (PMC11320443; doi:10.1016/j.heliyon.2024.e34881)
Supplement: Multimedia component 1 [file mmc1.pdf]

SUPPLEMENTARY DATA FOR

The Amorphization of Crystalline Silicon by Ball Milling

Roby Gauthier[a], B. Scott[a], J. Craig Bennett[b], Mina Salehabadi[a], Jun Wang[a], Tariq Sainuddin[a], and M.N. Obrovac*[a]

[a]Department of Chemistry, Dalhousie University, Halifax, N.S., B3H 4R2 Canada
[b]Department of Physics, Acadia University, Wolfville, N.S., B4P 2R6, Canada

* - corresponding author: mnobrovac@dal.ca

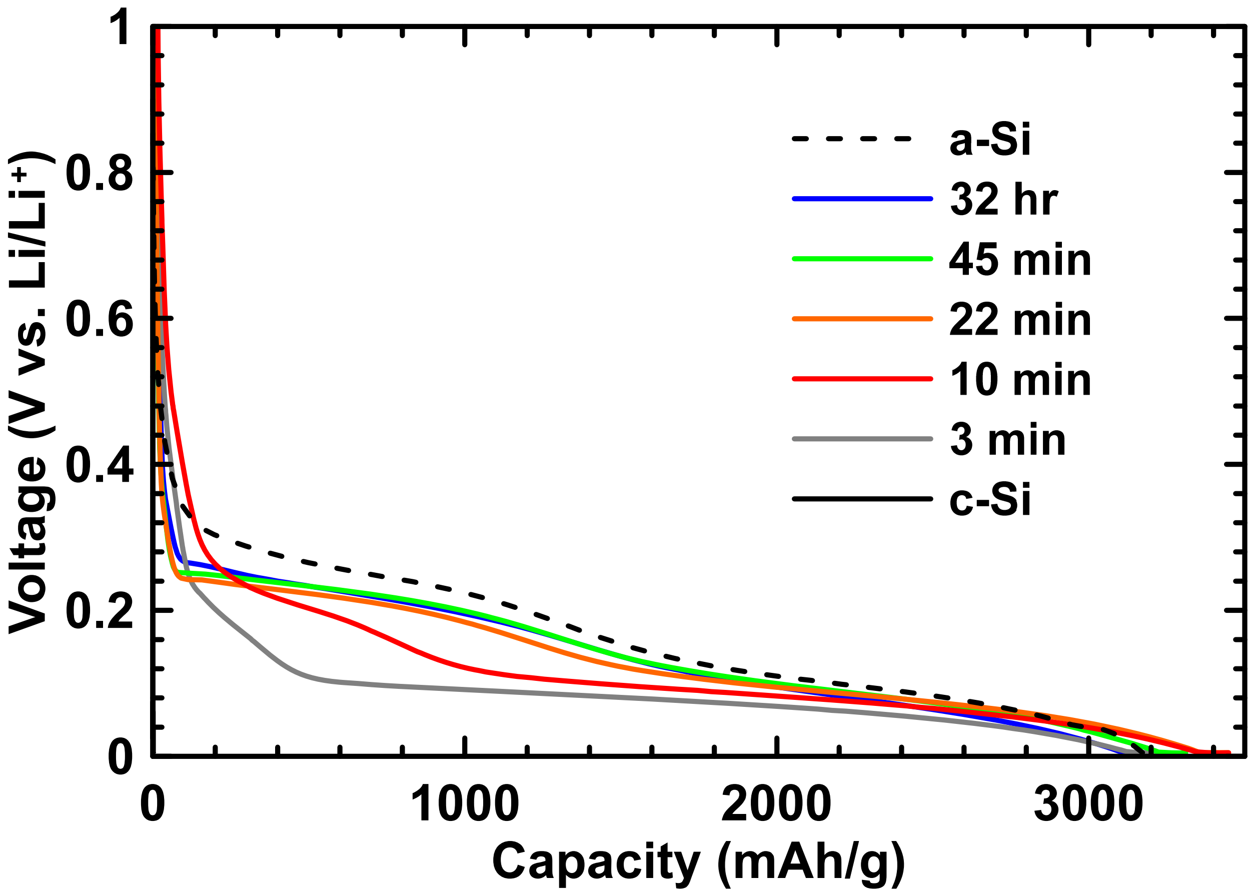

Figure S1 Discharge voltage curves of the silicon half-cells used in this study.
